# Diets of denning female Pacific martens vary with the developmental stage of their kits

**DOI:** 10.1002/ece3.5179

**Published:** 2019-04-29

**Authors:** Keith M. Slauson, William J. Zielinski

**Affiliations:** ^1^ USDA Forest Service Pacific Southwest Research Station Arcata California

**Keywords:** carnivore, denning, diet, marten, Martes, predation, prey

## Abstract

1. Food resources can be a limiting factor and natural and anthropogenic influences that alter the abundance of food resources can affect population performance and persistence. Reproduction in mammals is energetically costly; therefore, understanding how food resources influence reproduction is essential, especially for species of conservation concern.
2. The objectives of this study were to characterize Pacific marten (*Martes caurina*) diets during the denning period and determine whether diets differed by sex or by phase of the denning period.
3. We used 943 scats to reconstruct sex‐specific diets of martens in the Sierra Nevada mountains during the denning period to evaluate sex‐specific hypotheses of predation patterns.
4. During the lactation phase, females preyed primarily on large‐sized prey (62.5% metabolizeable energy) 5.7‐times more than males. This likely optimized both energy gain and minimized time spent away from dependent young. During the weaning phase, females preyed primarily on medium‐ (90–200 g) and large‐sized prey (87.7% metabolizeable energy). During the predispersal phase, females exhibited a 4.7‐fold increase in use of small‐sized (<50 g) prey during the time kits are learning to hunt.
5. Male overall diet and predation patterns appear to fit an optimal foraging strategy that is influenced primarily by prey profitability and abundance, with males preying primarily on medium‐sized prey, closest to meeting their lower energetic needs. In contrast, females appear to fit the predictions for a central place forager that is also influenced by prey profitability, but also the increased energetic and maternal demands of denning, leading them to use larger prey than males over the phases of the denning period when kits are growing. We hypothesize that switching to smaller prey is related to females assisting their kits in developing hunting skills and experience that may increase their chances of survival once they disperse.

## 1 INTRODUCTION

The influence of biotic and abiotic factors on population dynamics have long been of fundamental interest to ecologists (Andrewartha & Birch, 1954; Gaillard et al., 2010). More recently, the influence of anthropogenic factors on population dynamics has become a focus as human activities have stressed many of the world's ecosystems (Vitousek, Mooney, Lubchenco, & Melillo, 1997). For many species, including carnivores, food resources are a primary limiting factor for population growth. For example, in the boreal forest ecosystems of the Neartic and Paleartic, annual variation in reproduction and population density of both mammalian (Canada lynx [*Lynx canadensis*], Mowat, Poole, & O'Donoghue, 2000; American marten [*Martes americana*], Thompson & Colgan, 1987) and avian carnivores (Northern goshawk [*Accipiter gentilis*], Doyle & Smith, 1994, Sulkava, Huhtala, & Tornberg, 1994) is associated with cyclical fluctuations in their primary prey: lagomorphs, galliformes, and sciurids. Variation in the density and vulnerability of prey resources can also affect the spatial distribution of carnivores and variation in their reproductive output. For example, long‐term reproductive success of African lions (*Panthera leo*) is associated with a landscape feature, river confluences (Mosser, Fryxell, Eberly, & Packer, 2009), where ungulate prey both occur at high densities and are most vulnerable to predation by lions (Hopcraft, Sinclair, & Packer, 2005). To understand how the biotic, abiotic, and anthropogenic factors affect population dynamics, through their influences on food resources, the first step is to determine which food resources affect survival and reproduction.

The Pacific marten (*Martes caurina*) occurs throughout the higher elevations of mountains in the western United States and Canada and is of management or conservation interest throughout much of its' range. In the southern portions of the Pacific marten range, the most significant declines in distribution have occurred (Zielinski, Slauson, Carroll, Kent, & Kudrna, 2001), including in the northern Sierra Nevada mountains of California and Nevada (Zielinski, Truex, Schlexer, Campbell, & Carroll, 2005). The Pacific marten is designated as a sensitive species by the United States Forest Service and a species of special concern by the California Department of Fish and Wildlife. Although survival appears to affect population growth more than fecundity (Buskirk, Bowman, & Gilbert, 2012), few studies have implicated food resources as a limiting factor for survival. Studies of untrapped North American marten populations have primarily identified predation as the major cause of mortality (Bull & Heater, 2001; McCann, Zollner, & Gilbert, 2010); however see Johnson, Fryxell, Thompson, and Baker (2009) on the potential for food to limit juvenile survival. Martens have a polygamous mating system, with females solely responsible for the care and raising of young. If female martens are similar to other mustelids, they may require more than double the daily energy intake of the larger‐bodied males during the denning season (e.g., fisher [*Pekania pennanti*], Powell, 1993). Male Pacific martens average ~1,000 g with females typically ~25% smaller at 750 g (Slauson, 2017). In other sexually dimorphic carnivores, females may either exhibit sex‐specific selection for more prey‐rich habitat patches, use more energy‐rich prey, or both during the period when they rear young (Arronsson et al., 2016; Breed, Bowen, McMillan, & Leonard, 2006). Previous work intimated that the density of female Pacific martens was greater where more of their home range was associated with patches of old forest (Chapin, Harrison, & Phillips, 1997; Slauson, 2017). In addition to providing more denning structures, these older forest types are also assumed to provide high‐quality habitat for specific prey that are important food for martens (e.g., sciurids; Carey, 1991, Waters & Zabel, 1995). Whether female association with this habitat type during the denning season is directly linked to a special set of prey resources is unknown.

Reproduction in mammals is both energetically expensive (Gittleman & Thompson, 1988) and requires substantial commitment of nutritional resources by females (Oftendall, 1985). Furthermore, the behaviors supporting the development of young, such as provisioning of food and thermoregulation, change over the course of the development of young, from birth to dispersal. In North American martens, females typically give birth to altricial kits in April and spend little time away from the natal den immediately prior to and within 1–2 weeks after parturition (Henry, Doherty, Ruggiero, & Sickle, 1997). Kits are entirely dependent on milk for food until they are ~6 weeks of age. Energetic requirements for pregnant carnivores begin to exceed maintenance energy levels shortly after active gestation begins and then rapidly increase energy demand during lactation, when energetic requirement can be 2‐ to 3‐times higher than maintenance levels, depending on the number of young in the litter (Loveridge, 1986; Powell, 1993). Field metabolic rates estimated for female American martens (*M. americana*) in winter suggests females require ~107 g of fresh prey per day in winter to meet maintenance requirements (Gilbert, Zollner, Green, Wright, & Karasov, 2009). Therefore, if energy requirements double during lactation, females will require >200 g of fresh prey per day during the peak of lactation.

In addition to substantial increases in energy needs during the lactation phase, den attendance is also greatest during this phase. Females are needed at dens to provide kits with milk, assistance with thermoregulation, protection from predators, and therefore, foraging bouts are necessarily of short duration (Henry et al., 1997; Kleef & Tydeman, 2009). Given the energy needs of mothers increase when time available for hunting decreases, we predict that denning females should be most selective about foraging areas and focus their hunting on the most prey‐rich portions of their home range. About 6 weeks after parturition, lactation ceases and females begin bringing kits solid food (Mead, 1994). Den attendance by female martens declines during the weaning phase and females spend more time foraging and on longer foraging bouts (Henry et al., 1997; Kleef & Tydeman, 2009). Body mass gain by kits is greatest during the weaning phase. Kits begin to emerge from dens at about 8 weeks of age and presumably within the next few weeks begin to accompany females on foraging bouts where they start to develop hunting skills. At 12–16 weeks of age, young martens begin to disperse (Jones, Raphael, Forbes, & Clark, 1997; Schmidt, 1943; Wynne & Sherburne, 1984). Over the course of the denning period, both the energetic demands and maternal behavioral needs required by female martens change and these changes are dependent on the stage of kit development.

For natural selection to act on foraging behavior, decision mechanisms must affect the forager's reproduction or survival (Ydenberg, Brown, & Stephens, 2007). In our study, we investigated diet composition during the period of the year when female marten diet choices affect both survival and reproduction while male diet choice affects primarily survival. While both males and females experience the similarly high energetic costs of foraging (Gilbert et al., 2009), female's also have the increased risks of reproductive failure from being away from the den to influence their prey selection. Although males and females likely share one goal of foraging, to maximize energy gain, alternative roles in reproduction mean that males and females fit alternative foraging models, a central place model for females who must return to their dens and an optimal foraging model for males which can forage and rest in any part of their home ranges during foraging bouts. While we did not measure the prey delivered to the kits, we assume the prey being consumed by the females were the same. This assumption is certainly true in the lactation phase as females must consume all prey to convert it to milk for the kits. For females, capable of only delivering a single typical prey item, the decision they face is on the minimum size of prey to capture and consume/deliver to the den implying a trade‐off between low selectivity (capture any prey) and high selectivity (capture only large prey) and the associated increase in use of time in transit (low selectivity) versus searching (high selectivity). Single‐loader models predict that foragers should select a higher minimum prey sizes when prey are more abundant and when they must travel greater distances to capture sites (Ydenberg et al., 2007). Without the need to return to a den, males have fewer constraints on prey selection and should vary their level of selectivity primarily by the relative energetic profitabilities and abundances of available prey taxa (Charnov, 1976; Ydenberg et al., 2007). The energetic profitability of prey is typically scaled by body mass (Thompson & Colgan, 1990), therefore on the basis of weight prey rankings for our study were as follows: hare > ground squirrel > Douglas squirrel > chipmunk>pocket gopher > mole>deer mouse and by prey size classes large (>200 g) > medium (90–200 g) > small (≤50 g) prey. If martens forage to maximize their rate of energy intake, then in general they should always eat top‐ranked prey that meet or exceed their daily energy requirements and only include lower ranked prey once top‐ranked prey become too rare.

The overall objective of this study was to characterize the diet composition of the Sierra subspecies (*M. c. sierrae*) of the Pacific marten during the denning period of the year and determine whether it differed by sex or by phase of the denning period. Because the energetic needs and behaviors differ most between the sexes during the denning period, we hypothesized that overall diet composition should also differ during that period and that female diets should be composed of more energetically profitable, large‐bodied, prey than male diets. However, although we did not measure prey abundance, our study took place over the transition from low (May) to high (June–July) prey abundance as the snowpack declined and new prey populations emerged and reproduction in most prey populations occurred (Keane, Morrison, & Fry, 2006). During May, when prey populations are least abundant we predicted that males should capture any prey of any size (exhibit low selectivity), consistent with the predictions of an optimal foraging model and females should capture primarily large‐bodied prey (exhibit high selectivity) due to the combination of higher energetic needs and the need to reduce overall time away from the den foraging, consistent with the predictions of a central place foraging model. During June and July, when new prey populations emerge, we predicted both males and females should switch to increasing the use of the most abundant, energetically profitable prey sizes (exhibit high selectivity), and proportionately reduce use of the least profitable prey sizes, consistent with the predictions for both optimal and central place foraging models, respectively.

## 2 METHODS

### 2.1 Study area

**Figure 1:**
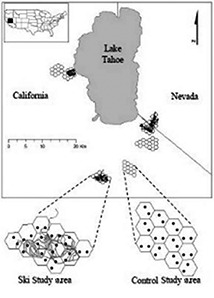
Location of the paired ski and control Pacific marten study areas and their sampling locations (dots) and ski runs (gray lines) in the Lake Tahoe Region of California and Nevada, USA (a). Habitat compositions of each paired ski and control study area (b) using the California Wildlife Habitat Relationships (WHR) classification and remotely sensed data available from the United States Forest Service Pacific Southwest Region Remote Sensing Lab's existing vegetation coverage (updated 2010). The WHR size classes (c) refer to the following tree diameter at breast height classes: 3 (15–28 cm), 4 (28–61 cm), and 5 (>61 cm)

We conducted our study in the Sierra Nevada mountains of California and Nevada, USA, on the Lake Tahoe Basin Management Unit and El Dorado National Forest, both administered by the United States Forest Service (Figure 1). Elevations ranged from about 2,000–3,000 m and the area was composed largely of forested habitats dominated by red fir (*Abies magnifica*), lodgepole pine (*Pinus contorta*), white fir (*Abies concolor*), western white pine (*P. monticola*), mountain hemlock (*Tsuga mertensiana*), and Jeffrey pine (*P. jeffreyi*). Over most of the 20th century the Lake Tahoe region has a dry‐summer continental climate with average temperatures ranging between 25.9 and 4.3°C and an average of 1,440 mm of precipitation falling predominantly as snow on the mesic western portions of the region where this study occurred. Scats were collected at six different study areas that ranged from 10 to 18 km^2^ in size and in total were 80 km^2^. Our scats were collected during the course of conducting a companion study to evaluate the effects of developed ski areas on martens (Slauson, Zielinski, & Schwartz, 2017).

### 2.2 Field collection of scats

During the months of May through July for 3 years (2009–2011) we collected scats opportunistically during a companion study that systematically live trapped martens to collect demographic parameters across the six study areas (Slauson, 2017; Slauson et al., 2017). During each month, a pair of ski and control study areas were live trapped using a trap density of 2‐traps/100 ha (Figure 1). Ten to forty traps were active during nearly every day of the month, providing a near‐continuous scat sampling effort during the sampling period (May–July). Knowing the sex and age of each individual, we trapped allowed us to associate age and sex to the scats collected from live traps and during processing. Scats containing significant amounts of trap bait (chicken) were excluded from analysis. We also excluded remains that were unlikely to be directly consumed for the purpose of acquiring energy, from grooming activities (e.g., marten hairs, ticks), and plant and soil debris (e.g., rocks, bark, conifer needles) commonly attached to the exterior of scats. Scats were stored dry in vials with desiccant.

The timing of our scat sampling overlapped the majority of the period just after kits are born until just prior to their annual dispersal. We defined three phases of the denning period, based on the developmental stage of the kits and likely implications this has on female energetics and foraging behavior: (a) *lactation* (weeks 0–6, mid‐April to May), (b) *weaning* (weeks 6–10, June), (c) *predispersal* (weeks 10–16, July to mid‐August). Diets were characterized temporally using these three phases of the denning period.

### 2.3 Identification of prey remains

Processing scats to identify remains followed a standard procedure previously described in Slauson and Zielinski (2017). We assigned prey remains to the most discriminating taxonomic level possible. We compared the characteristics of scat component groups to reference collection materials for hair, scales (reptiles), feathers, and skeletons (mammals). We also used published and unpublished keys to identify guard hairs (Adorjan & Kolenosky, 1969; Mayer, 1952; Moore, Spence, & Dugnolle, 1974), and field guides (Sibley, 2000) to identify feathers. Identifications of mammals typically occurred using skeletal elements, usually teeth and claws, and guard‐hair characteristics.

### 2.4 Summary of marten diet and statistical comparisons of diet by groups

Our primary metric for describing the marten diet was in terms of Proportion of Metabolizable Energy (PME), which is the least biased method and reflects the fundamental currency of consumption (Slauson & Zielinski, 2017). PME is the proportion of all the metabolizable energy represented by a single prey taxon. We categorized prey items in two ways: (a) taxonomically (b) by body size, using small (≤50 g), medium (90–200 g), and large (≥225 g) to define prey size categories that are relevant to the daily energetic needs of martens (Slauson & Zielinski, 2017). We applied the criterion of Trites and Joy (2005) to determine which comparisons had sufficient sample sizes to detect moderate effect sizes (≥0.30) with *α* = 0.05 and statistical power (1 − β) ≥ 0.80. They found that at least 59 scats were needed to distinguish prey species representing >5% frequency of occurrence in the overall diet but that at least 94 scats were necessary to make statistically valid comparisons between scat sample groups (e.g., sex, study areas). We first compared the overall, May–July diet composition by sex. We then compared changes in diet composition during each of the three phases of the denning period (lactation, weaning, predispersal) and overall diets between ski versus control areas. To limit the number of prey categories used in each comparison, we only used prey species representing ≥5% of PME. We used contingency table tests to identify significant differences and used Fisher's exact tests in program R (version 3.2.1; www.r-project.org). We compared PME for denning period phase and study area using these same methods. We controlled for our overall experimental‐wise error rate, due to conducting multiple statistical tests on the same dataset, using the Bonferroni correction procedure:

$$H_{i}\;\text{is rejected if}\;p_{i}\leq \alpha /m$$

where *H_i_* is the *i*th hypothesis to be tested, *p_i_* the *p*‐value for the *i*th statistical test, *α* = 0.05, and *m* the total number of hypotheses tested. We evaluated three hypotheses using two categorizations of our dataset, resulting in an overall experimental‐wise *α* = 0.05/6 = 0.008. Our methods adhered to the American Society of Mammalogists guidelines for use of live animals in research (Sikes, 2016).

### 2.5 Niche breadth and diet overlap

We followed the framework of De Cáceres, Sol, Lapiedra, and Legendre (2011) for estimating niche metrics using the resemblance between qualitative resources, the fact that some resources are more closely related in size and energy content than others. Calculations using this method have greater accuracy in their estimates and minimize the bias caused by the way resources are subdivided. To estimate diet niche breadth, we used the Euclidean diversity coefficient (Champely & Chessel, 2002) using the PME contributed by each prey item, described as:

$$B_{D}=\sum_{l=1}^{m}\left[\sum_{j=1}^{r}\cdot f_{j}{\left(x_{\mathrm{jl}}-{\bar{x}}_{1}\right)}^{2}\right]$$

where niche breadth (*B*) conditional on distance matrix *D*, where each element *d_jk_* contains the body size distance between pairs of prey taxa *j* and *k*, is the sum of the products of the vector of relative resource use (*f_i_*) and the squared distance(s) between the *j*th resource position (*x_jl_*) and the average resource position on the *l*th axis of the resource space (De Cáceres et al., 2011).

Niche breadth estimates were calculated by sex for the overall diet and for each month of the denning period; each niche breadth estimate included calculation of a 95% confidence interval using the R package “indicspecies” (version 1.7.6).

To estimate the overlap in prey use among the sexes during the denning period, we used the Renkonnen index (*P*; Krebs, 1999), described as:

$$P=\Sigma \;minimum(pM_{i},pF_{i})$$

where *P* is the percent similarity between the male and female samples, *pM_i_* the percentage of prey item *i* in the male sample and *pF_i_* the percentage of prey item *i* in the female sample, using the PME of prey items in our samples.

## 3 RESULTS

### 3.1 Overall diet

**Figure 2:**
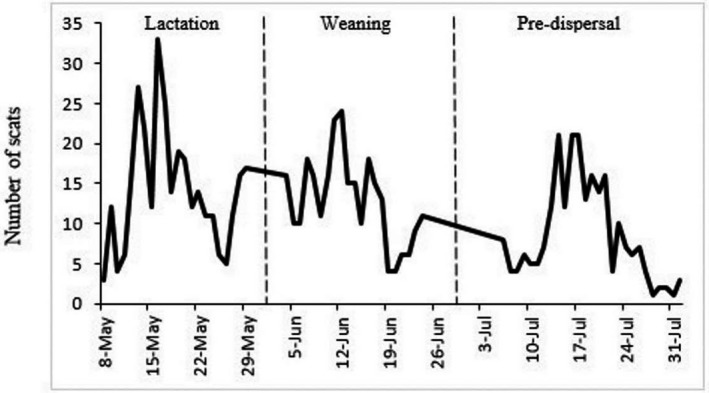
Daily collection rates of a total of 943 scats used for diet reconstruction from Pacific Martens from 2009 to 2011 in the Lake Tahoe region of California and Nevada

From May to July of 2009–2011, we collected a total of 1,333 scats (Figure 2), 390 of which included primarily trap bait and were excluded from analysis resulting in a final sample of 943 scats from 79 individual martens (52M:27F). The diet during the denning period included 5 classes of prey (Table 1) with mammals far more common (93% of ME) than all other classes. Fifteen taxa of mammals were found in the diet but 6 taxa including 3 Sciurids (44.9% ME), broad‐handed mole and montane pocket gopher (25.2% ME), and 1 Cricetid (11.4% ME) accounted for 80.5% of all ME (Table 1).

**Table 1:** Overall and sex‐specific proportions of metabolizable energy for prey items of Pacific martens (*Martes caurina*) from 2009 to 2011 in the Lake Tahoe region of California and Nevada

Taxa	Frequency (*n*)	Proportion of metabolizable energy				
		Overall (*n* = 847)	Male (*n* = 615)	Female (*n* = 232)	Denning female (*n* = 181)	Yearling female (*n* = 51)
Mammalia	888	93.3	91.8	93.3	92.7	94.8
*Scapanus latimanus*	96	9.4	12.0	1.9	2.2	0.7
*Lepus americanus*	34	4.9	4.9	4.9	6.0	0.0
Rodentia	781	78.7	74.8	89.2	83.5	94.1
Sciuridae	395	46.6	40.9	60.7	66.7	37.4
*Eutamias*spp.	156	15.6	15.9	17.5	16.9	22.1
*Tamiasciurus douglasii*	98	12.1	7.0	28.2	31.7	13.8
*Spermophilus lateralis*	125	17.2	16.2	15.0	18.1	1.5
*Thomomys monticola*	138	15.8	18.1	8.4	6.2	20.4
Cricetidae	253	16.3	15.8	20.2	16.8	36.3
*Peromyscus*spp.	191	11.4	10.7	16.2	12.7	33.0
Aves	133	6.7	8.1	3.2	1.1	5.3
Medium (40–200 g)	23	3.4	6.7	2.6	0.4	4.9

Note

Frequency (*n*) counts for each prey item represent the total number of scats in which it was identified. Species <5% PME: *Sorex* sp., *Ochotona princeps*, *Aplodontia rufa*, *Glaucomys sabrinus*, *Marmota flaviventris*, *Neotoma cinerea*, *Zapus princeps*, *Microtus longicaudus*, small birds (<40 g), large birds (>200 g), *Sceloporus* sp., Insecta, conifer seeds.

### 3.2 Sex‐specific overall diet comparisons during the denning period

**Figure 3:**
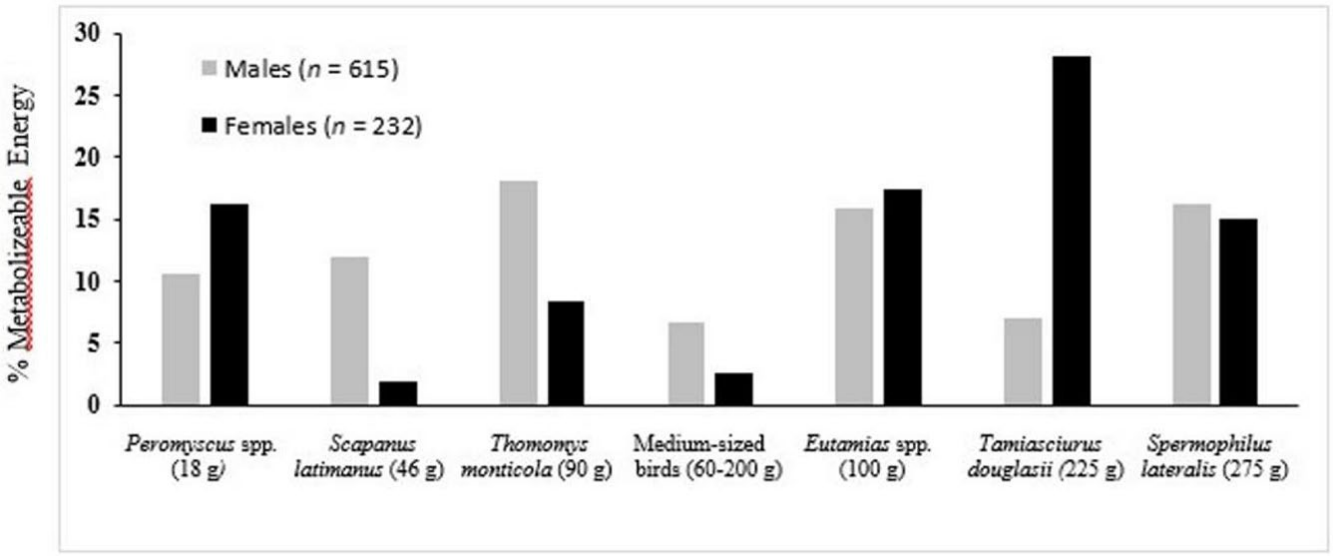
Overall dietary differences for prey taxa contributing >5% of metabolizable energy to the diets of male and female Pacific martens (*Martes caurina*) using 847 scats during the 2009–2011 denning seasons (May–July) in the Lake Tahoe region of California and Nevada

A total of 615 scats were from males and 232 scats were from females, sufficient sample sizes for making diet comparisons between the sexes (Trites & Joy, 2005). Overall diet composition between males and females, using the 7 prey taxa representing >5% ME for either sex (Table 1), was significantly different during the denning period (Pearson's *χ* ^2^ test = 68.1, *df* = 6, *p* < 0.0001). Overall diet niche breadth, considering the top 16 prey taxa, was significantly narrower for females than males and overall diet overlap, based on taxonomy of prey remains, was 53% (Table 2). Greater than twofold differences in the PME for primary prey species (>5% ME) between males and females occurred for 3 of the 7 prey taxa (Figure 3). Pocket gophers and moles represented 2.9 times greater PME for males (30.1% ME) than females (10.3% ME) and Douglas squirrels represented 4.0 times greater PME for females (28.2% ME) than males (7.0% ME; Table 1, Figure 3). Overall, the female diet during the sample period (May–July) represented fewer taxa than did the male diet, with the top 5 ranked taxa representing 85.3% ME, dominated by 3 sciurids (60.7% ME, Table 1, Figure 3). The top 7 ranked taxa in the male diet represented 86.6% ME, and the male diet was more evenly dominated by both sciurids (39.1% ME) and moles and pocket gophers (30.1% ME; Table 1).

**Table 2:** Overall and monthly diet composition, expressed as proportions of metabolizable energy (PME) of prey taxa grouped by body size (g), for male and denning female Pacific martens (*Martes caurina*) from 2009 to 2011 in the Lake Tahoe region of California and Nevada

	PME for prey body sizes		
	<50 g	90–120 g	>200 g
Male			
Overall	27.1	42.2	29.6
Lactation	46.4	40.1	11.7
Weaning	23.7	38.7	35.5
Predispersal	10.2	48.1	37.1
Denning female			
Overall	19.7	23.6	56.6
Lactation	13.9	18.4	66.1
Weaning	12.4	34.9	52.8
Predispersal	47.7	23.4	29.6
Nondenning female			
Overall	37.5	47.4	15.2

In respect to prey size, estimates of PME suggested that, across the denning period, females killed large prey (>200 g) almost twice as frequently as did males (48.8% vs. 29.6% ME, respectively). In contrast, the male diet during this period included 1.5‐times as many medium‐sized prey (40–200 g) than the female diet (28.5% vs. 42.4% ME, respectively). However, neither the PME of large prey nor the PME of medium‐sized prey were statistically different for males versus females (Pearson's *χ* ^2^ = 8.2, *df* = 2, *p* = 0.02). Overall diet niche breadth, measured by body size, was narrower for females compared to males (Table 3).

**Table 3:** Estimates of niche breadth (De Cáceres et al., 2011) and Renkonnen's index of diet overlap (Krebs, 1999) for Pacific martens (*Martes caurina*) from 2009 to 2011 in the Lake Tahoe region of California and Nevada

Metric	Sex	Niche breadth				Diet overlap				
		Overall	Lactation	Weaning	Predispersal		Overall (%)	May (%)	June (%)	July (%)
Taxon	Male	0.27 (0.25–0.29)	0.13 (0.09–0.17)	0.15 (0.11–0.19)	0.18 (0.13–0.22)		53	48	55	54
	Female	0.35 (0.33–0.38)	0.21 (0.15–0.27)	0.26 (0.19–0.32)	0.35 (0.29–0.39)					
Body size	Male	0.25 (0.18–0.32)	0.13 (0.09–0.17)	0.15 (0.11–0.19)	0.17 (0.04–0.26)	Small (≤50 g)	21	14	23	12
						Medium (90–200 g)	29	23	36	23
						Large (>200 g)	30	12	36	27
	Female	0.33 (0.27–0.37)	0.20 (0.15–0.26)	0.26 (0.20–0.31)	0.33 (0.25–0.37)	Total overlap	79	49	94	63

Of the total sample of scats from females (*n* = 232), 21.9% (*n* = 51) were from nondenning yearling females. We explicitly tested whether the diets of denning and nondenning females differed. Indeed, the overall diet composition between denning and nondenning females, using the 6 prey taxa representing >5% ME for either sex (Table 1), was significantly different during the denning period (Pearson's *χ* ^2^ test = 44.4, *df* = 5, *p* < 0.0001). In addition, the PME for prey size classes was significantly different between denning and nondenning females (Pearson's *χ* ^2^ test = 37.4, *df* = 2, *p* < 0.0001). Denning females used large‐sized prey 3.7‐times more frequently than nondenning females (56.6% vs. 15.2%, respectively) and medium and small prey 2.0‐ and 1.9‐times less, respectively (Table 1). The diets of nonreproducing, yearling females were overall more similar to the diet of males. For this reason, they were excluded from subsequent analyses of diet composition by the phase of the denning period. When PME for prey size classes were compared between males and denning females over the denning period (Table 2), the differences became significant (Pearson's *χ* ^2^ test = 14.8, *df* = 2, *p* < 0.0006).

### 3.3 Comparisons of sex‐specific diets across phases of the denning period

**Figure 4:**
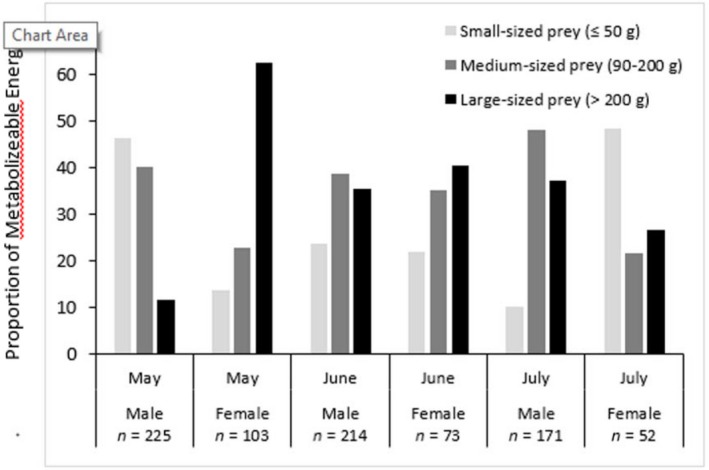
Sex‐specific predation phenology of Pacific martens (*Martes caurina*) from 2009 to 2011, based on diet reconstruction using 796 scats (610M:187F), during the three energetic phases (lactation, weaning, predispersal) of the denning period (May–August) in the Lake Tahoe region of California and Nevada

We collected 328 (225M:84F) scats during the lactation phase, 287 (214M:47F) scats in the weaning phase, and 223 (171M:46F) scats in the predispersal phase of the denning season. The diet of male martens, using the 7 taxa with the highest MEs, differed significantly from that of females during the lactation phase (Pearson's *χ* ^2^ = 67.5, *df* = 6, *p *≤ 0.0001), during the weaning phase (Pearson's *χ* ^2^ = 46.5, *df* = 6, *p *≤ 0.0001), and during the predispersal phase (Pearson's *χ* ^2^ = 55.4, *df* = 6, *p *≤ 0.0001; Table 4). Sex‐specific diets, using the three body size categories, also were significantly different between males and females for the lactation (Pearson's *χ* ^2^ = 57.2, *df* = 2, *p *≤ 0.0001) and predispersal phases (Pearson's *χ* ^2^ = 36.5, *df* = 2, *p *≤ 0.0001), but not during the weaning phase (Pearson's *χ* ^2^ = 7.01, *df* = 2, *p* = 0.03, Table 2). During the lactation phase, males preyed on more small‐ (3.4‐times greater) and medium‐sized (2.2‐times greater) prey than females (Figure 4) and these differences were primarily due to males predating on white‐footed mice (29.9% vs. 10.8% ME, respectively) and moles and pocket gophers (39.2% vs. 6.3% ME, respectively) at rates 2.8‐ to 6.2‐times greater than females, respectively (Table 3). During the lactation phase, females preyed on large‐sized prey 5.7‐times more than males and this was primarily due to females preying 11.7‐times more on Douglas' squirrels than males (44.6% vs. 3.8% ME, respectively).

**Table 4:** Diet compositions for male and female Pacific martens (*Martes caurina*) during each phase of the denning period from 2009 to 2011 in the Lake Tahoe region of California and Nevada, considering only those species represented by >5% PME (Species <5% PME: *Sorex* sp., *Ochotona princeps*, *Aplodontia rufa*, *Glaucomys sabrinus*, *Marmota flaviventris*, *Neotoma cinerea*, *Zapus princeps*, small birds (<50 g), medium birds (50–200) large birds (>200 g), *Sceloporus* sp., Insecta, conifer seeds)

Taxa	Proportion of metabolizable energy					
	Lactation phase		Weaning phase		Predispersal phase	
	Males (*n* = 225)	Denning females (*n* = 84)	Males (*n* = 214)	Denning females (*n* = 47)	Males (*n* = 171)	Denning females (*n* = 46)
Mammalia						
*Scapanus latimanus*	15.4	1.7	17.6	0.0	7.4	8.3
*Lepus americanus*	2.2	10.0	6.8	2.8	<1	0.0
Rodentia						
Sciuridae						
*Eutamias*spp.	15.7	13.8	17.8	31.8	23.5	1.4
*Tamiasciurus douglasii*	3.8	44.6	9.6	10.0	13.4	24.6
*Spermophilus lateralis*	5.7	10.1	16.1	41.4	18.6	4.6
*Thomomys monticola*	23.8	4.6	15.4	1.5	23.5	20.6
Cricetidae						
*Peromyscus*spp.	29.9	10.8	3.5	11.5	2.0	21.7
*Microtus longicaudus*	<1	1.7	2.5	1.6	<1	14.6

During the weaning phase, females preyed primarily (87.7% ME) on large (52.8% ME, 1.5‐times more than males) and medium prey (34.9% ME; Table 2), but males increased their use of these prey size classes but continued to use small‐size prey 1.9‐times more than females (Table 2). During the weaning phase, females preyed primarily (73.1% ME) on large (*Spermophilus lateralis*, 41.4% ME) and medium‐sized (*Tamias*, 31.8% ME) sciurids while males used sciurids (43.5% ME) and moles and pocket gophers (33.0% ME) at similar rates (Table 4). During the predispersal phase, predation patterns based on body size switched, with females taking 4.7‐times more small prey, 50% fewer medium‐ and 25% fewer large‐sized prey than males (Table 2, Figure 4). These differences in predation patterns during the preweaning phase are primarily due to females reducing their use of ground‐dwelling sciurids from 60.9% to 5.2% from the weaning to predispersal phases, while increasing their predation rates on white‐footed mice and long‐tailed voles 13.6‐times more than males over this same transition period (Figure 4).

## 4 DISCUSSION

Differences in the diets of males and females were greatest when compared between each denning phase and least when aggregated across all phases of the denning season. Overall, PME for any individual prey taxa or prey body size category did not differ more than 27% between the sexes. However, females had a narrower diet breadth and the largest differences were that, despite being ~25% smaller on average than males, female martens preyed more on large‐sized prey (56.6% ME vs. 29.6%) and less on small‐ (19.7% vs. 27.1% ME) and medium‐sized (23.6% vs. 42.2% ME) prey than males during the denning season. This was consistent with our overall hypothesis that female diets should be composed of more energetically profitable prey than males due to the increased energetic demands required by females to raise young.

There are important changes in marten diets during the denning season that are potentially influenced by the changing energetic needs of females, changes in maternal responsibilities, and how each sex responds to changes in overall prey abundances.

During lactation, the most energetically demanding phase for females of the denning period, diet overlap between the sexes was lowest (Table 3), considering either prey species or size overlap. More specifically, during the lactation phase, female marten diets exhibited the highest selectivity during the period of lowest overall prey abundance. Female diets were dominated by large prey which they consumed at >5 times the rate as males (Figure 4). The Douglas' squirrel was the dominant large‐bodied prey species in the diet of females during the lactation phase and was preyed upon >11 times more frequently than by males. Furthermore, females are preying on large‐bodied prey at the time of the year (early spring) when Douglas' squirrel, ground squirrel, and hare populations are likely at their lowest, due to overwinter mortality, hibernation (ground squirrels), and prior to the birthing pulse Keane et al. (2006). The high selectivity, capturing primarily large‐sized prey, exhibited by female martens is consistent with the predictions of a central place forager (Ydenberg et al., 2007), which minimizes the time spent foraging, and away from the den, by capturing primarily single prey items meeting or exceeding their total daily energy needs despite their rarity. In contrast, males exhibited low selectivity, catching prey of any size, during the time when prey resources were least abundant and is consistent with the predictions of an optimal foraging model (e.g., Charnov) that low profitability small prey are only utilized when larger prey become rare.

Once the lactation phase transitioned into the weaning phase and overall prey resources increased due to the emergence of prey that hibernate or were active but under snowpacks, differences between the diets of males and females decreased. Use of body size classes became most similar between the sexes, but females continued to prey on more larger‐sized prey (52.8% vs. 35.5% ME) and less on small‐sized prey (12.4% vs. 23.7% ME) than males. During this phase, males continued to prey on moles and pocket gophers at a higher rate than females while females continued to eat primarily sciruids at higher rates than males. From the lactation to weaning phase, females switched from foraging predominantly on Douglas' squirrels to foraging predominantly on larger golden‐mantled ground squirrels and smaller chipmunks. This is also consistent with our prediction that during the weaning phase, when kits rapidly increase their body masses by consuming solid prey, females continue to the most abundant large‐bodied prey to meet the high energetic needs for providing the rapidly growing kits solid food. From the lactation to weaning phase, males increased their use of large prey and decreased their use of small prey. We hypothesize that these switches in predation on sciurid species by females and larger prey by males were both likely functional responses to increases in their respective abundances. Females, switched primary prey types by increasing only the next highest (ground squirrel) or next lowest (chipmunk) ranked prey while still occasionally preying on the reduced abundance of Douglas' squirrels from heavy predation during the lactation phase, thereby maintaining the majority (87.7% vs. 84.5% ME) of their energy intake from the medium‐ and large body‐sized prey. This is consistent with the predictions for maintaining foraging efficiency from a central place forager model. Males, decreased their use of the least profitable prey, small body sizes by 22.7% while increasing the most profitable body sizes by nearly the same amount, 23.8% and exhibiting little change (2.6%) for medium body sizes. This is consistent with the predictions from an optimal foraging model, where once abundances of higher ranked prey increase, decreased use of the lowest ranked prey should occur first.

As the weaning phase transitioned into the predispersal phase, differences between the diets of males and females again increased. Males increased their use of medium‐sized prey, increasing their predation rates mainly on ground‐dwelling sciurids when the young of these species are available and decreased their use of small‐sized prey. And again, these changes in diet were consistent with the predictions of an optimal forager, which when use increases for a higher ranked prey, decreased use should first occur in lower ranked prey. Females had the opposite and more extreme shift, decreasing their use of both medium‐ and large‐sized prey and increasing their use of small prey. The magnitude of changes for individual prey taxa were modest for males, 10.2% for one taxa, but for females changes for six species ranged from 10% to 37% (Table 4) and the increased use of small prey (35.3% ME) represented the largest change in the use of any body size by either sex across all phases of the denning period (Table 2). During the predispersal phase, females preyed most frequently (47.7% ME) on small‐sized prey and small prey and pocket gophers combined (68.3%) that are either terrestrial or fossorial. This change would only be consistent with the prediction from a central place forager model, if other medium‐sized and all large‐sized prey populations became so rare that it now became energetically favorable to switch to predominantly small prey. This would appear plausible if males showed a similar switch, as both sexes did when prey abundances increased from the lactation to weaning phases, but males maintained using prey predominantly in the medium‐ and large body size classes during this time. This could also be plausible if females had simply exhausted the medium‐ and large‐sized prey populations in the smaller portions of their annual home ranges they typically use during the denning period, whereas males continue to utilize their entire home ranges and therefore do not experience such resource depression. However, that too seems unlikely as during the predispersal phase kits become mobile and are capable of foraging with their mothers, allowing for expanded use of their home ranges during this phase. We suggest that this change in prey use may be motivated by another factor than foraging efficiency, the need to assist their kits in developing hunting skills. We hypothesize that females shift to smaller and more terrestrial prey during the preweaning phase to help the kits to begin developing the skills to find, stalk, and attack prey prior to dispersal. Yearling females in our study appear to also signal the greater importance of small prey in their diets (37.5% ME) during the denning period and starvation has been implicated in reduced juvenile survival elsewhere for martens (Johnson et al., 2009). This behavioral decision offers a trade‐off from simply maximizing foraging efficiency and increasing survival of young once they disperse that can potentially be influenced by natural selection.

Reproduction is often the most energetically demanding portion of a mammal's life cycle; therefore, it is critical to understand how important food resources supporting this activity are related to the habitats they used. The density of female martens in our study area has been shown to be most related to large patch sizes of the oldest forest habitats available (Slauson, 2017). Relationships between animals and the habitats they select often are multidimensional, and relate to how critical life history needs are met by features that occur in those habitats. Overall, sciurids were the source of the majority of the energy used during the denning period and during the most energetically demanding phases, lactation and weaning, of the denning period. The Douglas' squirrel appears to be a critically important prey species during the beginning of the denning season when prey resources are at their lowest (e.g., Keane et al., 2006), providing nearly half of the ME during the lactation phase. The importance of Douglas squirrels as a source of energy early in the reproduction period is similar for the similar sized Northern goshawk (*Accipiter gentilis*) in our study region (Keane et al., 2006). Douglas squirrel abundance varies in concordance with conifer cone crop production (Smith, 1968; Sullivan & Sullivan, 1982) and both the magnitude and frequency of cone production increases with increasing size of conifers (Burns & Honkala, 1990; Fowells & Schubert, 1956). Accordingly, Douglas' squirrels are typically most abundant in mesic late‐successional forests, where the resources they require, conifer seed crops, truffles, and large‐diameter logs and stumps for cache sites are most abundant (Buchanan, Lundquist, & Aubry, 1990; Hallett, O'Connell, & Maguire, 2003; Smith, Anthony, Waters, Dodd, & Zabel, 2003). The other dominant sciurid prey taxa, golden‐mantled ground squirrel and chipmunks, utilize most of the same food resources as Douglas's squirrels and whose abundances are typically highest where these food resources are most abundant (reviewed in Woodbridge, Hansen, & Dunk, 2012). Therefore, selection by females for large patches of the oldest forest available is likely to be because these patches provide: (a) the structural resources essential for reproduction—large‐diameter live and dead trees with cavities to serve as dens (Ruggiero, Pearson, & Henry, 1998) and (b) abundant prey resources in the body size classes that best support each of the phases of the denning period. Our diet findings suggest a direct link between the habitat female martens select and the prey resources they require during the most energetically demanding phases of the denning period. These findings are consistent with previous research that found martens using dens in habitat with the most abundant prey resources (Pearson & Ruggiero, 2001), suggesting an important relationship between the proximity of suitable den structures to the amount of habitat necessary to support the prey resources necessary for successful reproduction.

Understanding how a species' feeding ecology relates to the habitats they use is both essential for understanding species–habitat relationships and for providing a sound basis for management. Both sexes preyed most on taxa in the size class that overlapped their daily energetic needs during the denning period: large‐sized prey for females and medium‐sized for males. Without the additional energetic demands of denning, male predation patterns over the denning period appeared to closely follow the use of the most energetically profitable prey taxa (size/abundance) as prey resources changed from low to high during the denning period. Male martens in our study area appear to be able to meet their much lower energetic needs by foraging in both mature and old forest habitat (Slauson, 2017) and by relying more on prey taxa associated with more open and nonforest habitat (moles and pocket gophers) conditions than females during the denning period. Female martens appear to meet the high energetic demands of reproduction by both selecting more energy‐rich habitat patches (old forest) and using larger, more energy‐rich, prey consistent with behaviors observed in other species of sexually dimorphic carnivores (Arronsson et al., 2016; Breed et al., 2006). Overall, our results are an extension of our earlier work which supported the hypothesis that martens should select as prey those with body sizes that most clearly approximate their daily energetic needs (Slauson & Zielinski, 2017). This previous work identified variation in seasonal diet that was based on the body size of the prey. Furthermore, our results presented here support the hypothesis that diet varies both overall with sex and by each phase of the denning period and is influenced by changes in abundance for both sexes and by the different energetic and maternal demands placed on females during denning. A consistent result from studies of the Pacific marten in California, including ours, is the preeminent role of sciurids in the diet during both the winter‐spring and summer‐fall periods (*M. c. sierrae*: Zielinski, Spencer, & Barrett, 1983, Hargis & McCullough, 1984, current study, K. Slauson pers. obs.; *M. c. humboldtensis*: Slauson & Zielinski, 2017), and especially for females during the denning period (*M. c. sierrae*: current study; *M. c. humboldtensis*: Slauson & Zielinski, 2017). The importance of sciruid prey for reproduction and survival in these conifer‐dominated ecosystems likely explains one critical link between the close association between Pacific martens and late‐successional forest habitats consistently found by studies conducted in this part of their range (Moriarty, Zielinski, & Forsman, 2011; Slauson, 2017; Slauson, Zielinski, & Hayes, 2007; Spencer, Barrett, & Zielinski, 1983).

## CONFLICT OF INTEREST

None declared.

## AUTHOR CONTRIBUTIONS

K. Slauson collected and processed scat samples, conducted all analyses, and developed the manuscript. B. Zielinski assisted with interpretation of analysis results and development of the manuscript.

## DATA ACCESSIBILITY

The data used in this manuscript will be publically archived upon publication at the U. S. Forest Service's Research Data Archive.

## References

[ece35179-bib-0001] Adorjan, A. S. , & Kolenosky, G. B. (1969). A manual for the identification of hairs of selected Ontario Mammals. Ontario Lands and Forests Department, Ontario, Canada. Research Report, 90, 1–64.

[ece35179-bib-0002] Andrewartha, H. G. , & Birch, L. C. (1954). The distribution and abundance of animals. Chicago, IL: University of Chicago Press.

[ece35179-bib-0003] Arronsson, M. , Low, M. , Lopez‐bao, J. V. , Persson, J. V. , Odden, J. , Linnell, J. D. , & Andren, H. (2016). Intensity of space use reveals conditional sex‐specific effects of prey and conspecific density on home range size. Ecology and Evolution, 6(9), 2957–2967. 10.1002/ece3.2032 27217946 PMC4863019

[ece35179-bib-0004] Breed, G. A. , Bowen, W. D. , McMillan, J. I. , & Leonard, M. L. (2006). Sexual segregation of seasonal foraging habitats in a non‐migratory marine mammal. Proceedings of the Royal Society of London B: Biological Sciences, 273(1599), 2319–2326. 10.1098/rspb.2006.3581 PMC1636079 16928634

[ece35179-bib-0005] Buchanan, J. B. , Lundquist, R. W. , & Aubry, K. B. (1990). Winter populations of Douglas' squirrels in different‐aged Douglas‐fir forests. Journal of Wildlife Management, 54, 577–581. 10.2307/3809351

[ece35179-bib-0006] Bull, E. L. , & Heater, T. W. (2001). Survival, causes of mortality, and reproduction in the American marten in northeastern Oregon. Northwestern Naturalist, 82, 1–6. 10.2307/3536640

[ece35179-bib-0007] Burns, R. M. , & Honkala, B. H. (1990). Silvics of North America. Vol. 1, conifers. Agricultural Handbook 654. Washington, DC: USDA Forest Service.

[ece35179-bib-0008] Buskirk, S. W. , Bowman, J. , & Gilbert, J. H. (2012). Population biology and matrix demographic modeling of American martens and fishers In Aubry K., Zielinski W. J., Raphael M., Proulx G., & Buskirk S. (Eds.), Biology and conservation of martens, sables, and fishers: A new synthesis (pp. 77–92). Ithaca, NY: Cornell University Press.

[ece35179-bib-0009] Carey, A. B. (1991). The biology of arboreal rodents in Douglas‐fir forests. In: Huff, M.A.; Holthausen, R.S.; Aubry, K.B. (technical coordinators). Biology and management of old‐growth forests. Gen. Tech. Rep. PNW‐GTR‐276 (pp. 1–46). Portland, OR: U.S. Department of Agriculture, Forest Service, Pacific Northwest Research Station.

[ece35179-bib-0010] Champely, S. , & Chessel, D. (2002). Measuring biological diversity using Euclidean metrics. Environmental and Ecological Statistics, 9, 167–177.

[ece35179-bib-0011] Chapin, T. G. , Harrison, D. J. , & Phillips, D. M. (1997). Seasonal habitat selection by marten in an untrapped forest preserve. The Journal of Wildlife Management, 61, 707–717. 10.2307/3802178

[ece35179-bib-0012] Charnov, E. L. (1976). Optimal foraging: Attack strategy of a mantid. The American Naturalist, 110(971), 141–151. 10.1086/283054

[ece35179-bib-0013] De Cáceres, M. , Sol, D. , Lapiedra, O. , & Legendre, P. (2011). A framework for estimating niche metrics using the resemblance between qualitative resources. Oikos, 120, 1341–1350. 10.1111/j.1600-0706.2011.19679.x

[ece35179-bib-0014] Doyle, F. I. , & Smith, J. M. (1994). Population responses of northern goshawks to the 10‐year cycle in numbers of snowshoe hares. Studies in Avian Biology, 16, 122–129.

[ece35179-bib-0015] Fowells, H. A. , & Schubert, G. H. (1956). Seed crops of forest trees in the pine region of California. USDA Forest Service Technical Bulletin No. 1150. USDA Forest Service, Pacific Southwest Research Station, Redding, CA.

[ece35179-bib-0016] Gaillard, J. M. , Hebblewhite, M. , Loison, A. , Fuller, M. , Powell, R. , Basille, M. , & Van Moorter, B. (2010). Habitat–performance relationships: Finding the right metric at a given spatial scale. Philosophical Transactions of the Royal Society B: Biological Sciences, 365, 2255–2265. 10.1098/rstb.2010.0085 PMC2894964 20566502

[ece35179-bib-0017] Gilbert, J. H. , Zollner, P. A. , Green, A. K. , Wright, J. L. , & Karasov, W. H. (2009). Seasonal field metabolic rates of American martens in Wisconsin. The American Midland Naturalist, 162, 327–334. 10.1674/0003-0031-162.2.327

[ece35179-bib-0018] Gittleman, J. L. , & Thompson, S. D. (1988). Energy allocation in mammalian reproduction. American Zoologist, 28(3), 863–875. 10.1093/icb/28.3.863

[ece35179-bib-0019] Hallett, J. G. , O'Connell, M. A. , & Maguire, C. C. (2003). Ecological relationships of terrestrial small mammals in western coniferous forests. Mammal Community Dynamics, 114, 120–156.

[ece35179-bib-0020] Hargis, C. D. , & McCullough, D. R. (1984). Winter diet and habitat selection of marten in Yosemite National Park. The Journal of Wildlife Management, 48, 140–146. 10.2307/3808461

[ece35179-bib-0021] Henry, S. E. , Doherty, E. C. , Ruggiero, L. F. , & van Sickle, W. D. (1997). Maternal den attendance patterns of female American martens In Proulx G., Bryant H. M., & Woodward P. M. (Eds.), Martes: taxonomy, ecology, techniques, and management (pp. 78–85). Edmonton, Canada: Provincial Museum of Alberta.

[ece35179-bib-0022] Hopcraft, J. G. C. , Sinclair, A. E. , & Packer, C. (2005). Planning for success: Serengeti lions seek prey accessibility rather than abundance. Journal of Animal Ecology, 74(3), 559–566. 10.1111/j.1365-2656.2005.00955.x

[ece35179-bib-0023] Johnson, C. A. , Fryxell, J. M. , Thompson, I. D. , & Baker, J. A. (2009). Mortality risk increases with natal dispersal distance in American martens. Proceedings of the Royal Society of London B: Biological Sciences, 276, 3361–3367. 10.1098/rspb.2008.1958 PMC2817161 19570789

[ece35179-bib-0024] Jones, L. C. , Raphael, M. G. , Forbes, J. T. , & Clark, L. A. (1997). Using remotely activated cameras to monitor maternal dens of martens In Proulx G., Bryant H. M., & Woodward P. M. (Eds.), Martes: taxonomy, ecology, techniques, and management. (pp. 329–349). Edmonton, Canada: Provincial Museum of Alberta.

[ece35179-bib-0025] Keane, J. J. , Morrison, M. L. , & Fry, D. M. (2006). Prey and weather factors associated with temporal variation in northern goshawk reproduction in the Sierra Nevada, California. Studies in Avian Biology, 31, 87.

[ece35179-bib-0026] Kleef, H. L. , & Tydeman, P. (2009). Natal den activity patterns of female pine martens (*Martes martes*) in the Netherlands. Lutra, 52(1), 3–14.

[ece35179-bib-0027] Krebs, C. J. (1999). Ecological methodology. Menlo Park, California: Addison Welsey Educational Publishers. Inc..

[ece35179-bib-0028] Loveridge, G. G. (1986). Bodyweight changes and energy intake by of cats during gestation and lactation. Animal Technology, 37, 7–15.

[ece35179-bib-0029] Mayer, W. V. (1952). The hair of California mammals with keys to the dorsal guard hairs of California Mammals. The American Midland Naturalist, 48, 480–512. 10.2307/2422262

[ece35179-bib-0030] McCann, N. P. , Zollner, P. A. , & Gilbert, J. H. (2010). Survival of adult martens in northern Wisconsin. Journal of Wildlife Management, 74(7), 1502–1507. 10.2193/2009-297

[ece35179-bib-0031] Mead, R. A. (1994). Reproduction in martes. Martens, sables, and fishers: Biology and conservation (pp.404–422).

[ece35179-bib-0032] Moore, T. D. , Spence, L. E. , & Dugnolle, C. E. (1974). Identification of the dorsal guard hairs of some mammals of Wyoming. Laramie, WY: Wyoming Game and Fish Department.

[ece35179-bib-0033] Moriarty, K. M. , Zielinski, W. J. , & Forsman, E. D. (2011). Decline in American marten occupancy rates at Sagehen Experimental Forest, California. The Journal of Wildlife Management, 75(8), 1774–1787. 10.1002/jwmg.228

[ece35179-bib-0034] Mosser, A. , Fryxell, J. M. , Eberly, L. , & Packer, C. (2009). Serengeti real estate: Density vs. fitness‐based indicators of lion habitat quality. Ecology Letters, 12(10), 1050–1060. 19708970 10.1111/j.1461-0248.2009.01359.x

[ece35179-bib-0035] Mowat, G. , Poole, K. G. , & O'Donoghue, M. (2000). Ecology of lynx in northern Canada and Alaska In Ruggiero L. F., Squires J. R., Buskirk S. W., Aubry K. B., McKelvey K. S., Koehler G., & Krebs C. J. (Eds.). Ecology and conservation of lynx in the United States (pp. 265–306). Boulder, CO: University of Colorado Press.

[ece35179-bib-0036] Oftendall, O. T. (1985). Pregnancy and lactation In Hudson R. J., & White R. G. (Eds.), The Bioenergetics of Wild Herbivores. (pp. 215–238) Raton, FL: CRC Press, Boca.

[ece35179-bib-0037] Pearson, D. E. , & Ruggiero, L. F. (2001) Test of the prey‐base hypothesis to explain use of red squirrel midden sites by American martens. Canadian Journal of Zoology, 79(8), 1372–1379. 10.1139/z01-090

[ece35179-bib-0038] Powell, R. A. (1993). The fisher: Life history, ecology, and behavior (2nd ed.). Minneapolis, MN: University of Minnesota Press.

[ece35179-bib-0040] Ruggiero, L. F. , Pearson, D. , & Henry, S. E. (1998). Characteristics of American marten den sites in Wyoming. The Journal of Wildlife Management, 62, 663–673. 10.2307/3802342

[ece35179-bib-0041] Schmidt, T. F. (1943). Monographs on the wild animals. (Vol. 10). G6ttigen, Germany: Institute Fiir Jagdkunde, University of G6ttingen.

[ece35179-bib-0042] Sibley, D. A. (2000). The Sibley guide to birds. New York, NY: Knopf Publishing.

[ece35179-bib-0043] Sikes, R. S. & the Animal Care and Use Committee of the American Society of Mammalogists (2016). 2016 guidelines of the American Society of Mammalogists for the use of wild mammals in research and education. Journal of Mammalogy, 97, 663–688. 10.1093/jmammal/gyw078 29692469 PMC5909806

[ece35179-bib-0044] Slauson, K. M. (2017). Linking landscape pattern to population process in a carnivorous mammal. Dissertation, Missoula, MT: University of Montana.

[ece35179-bib-0045] Slauson, K. M. , & Zielinski, W. J. (2017). Seasonal specialization in diet of the Humboldt marten (*Martes caurina humboldtensis*) in California and the importance of prey size. Journal of Mammalogy, 98, 1697–1708. 10.1093/jmammal/gyx118

[ece35179-bib-0046] Slauson, K. M. , Zielinski, W. J. , & Hayes, J. P. (2007). Habitat selection by American martens in coastal California. Journal of Wildlife Management, 71(2), 458–468. 10.2193/2004-332

[ece35179-bib-0047] Slauson, K. M. , Zielinski, W. J. , & Schwartz, M. K. (2017). Ski areas affect Pacific marten movement, habitat use, and density. The Journal of Wildlife Management, 81, 892–904. 10.1002/jwmg.21243

[ece35179-bib-0048] Smith, C. C. (1968). The adaptive nature and social organization of tree squirrels *Tamiasciurus* . Ecological Monographs, 38, 31–63. 10.2307/1948536

[ece35179-bib-0049] Smith, W. P. , Anthony, R. G. , Waters, J. R. , Dodd, N. L. , & Zabel, C. J. (2003). Ecology and conservation of arboreal rodents of western coniferous forests. Mammal community dynamics. Management and conservation in the coniferous forests of Western North America (pp. 157–206). Cambridge, UK: Cambridge University Press.

[ece35179-bib-0050] Spencer, W. D. , Barrett, R. H. , & Zielinski, W. J. (1983). Marten habitat preferences in the northern Sierra Nevada. The Journal of Wildlife Management, 47(4), 1181–1186. 10.2307/3808189

[ece35179-bib-0051] Sulkava, S. , Huhtala, K. , & Tornberg, R. (1994). Regulation of Goshawk Accipiter gentilis breeding in Western Finland over the last 30 years In Mayberg U. B. & Chancellor R. D. (Eds.) Raptor conservation today (pp. 67–76). Flossmoor, IL: Pica Press.

[ece35179-bib-0052] Sullivan, T. P. , & Sullivan, D. S. (1982). Population dynamics and regulation of the Douglas squirrel (*Tamiasciurus douglasii*) with supplemental food. Oecologia, 53, 264–270. 10.1007/BF00545675 28311121

[ece35179-bib-0053] Thompson, I. D. , & Colgan, P. W. (1987). Numerical responses of martens to a food shortage in northcentral Ontario. Journal of Wildlife Management, 51, 824–835. 10.2307/3801748

[ece35179-bib-0054] Thompson, I. D. , & Colgan, P. W. (1990). Prey choice by marten during a decline in prey abundance. Oecologia, 83(4), 443–451. 10.1007/bf00317193 28313176

[ece35179-bib-0055] Trites, A. W. , & Joy, R. (2005). Dietary analysis from fecal samples: How many scats are enough? Journal of Mammalogy, 86, 704–712.

[ece35179-bib-0056] Vitousek, P. M. , Mooney, H. A. , Lubchenco, J. , & Melillo, J. M. (1997). Human domination of Earth's ecosystems. Science, 277, 494–499. 10.1126/science.277.5325.494

[ece35179-bib-0057] Waters, J. R. , & Zabel, C. J. (1995). Northern flying squirrel densities in fir forests of northeastern California. Journal of Wildlife Management., 59, 858–866. 10.2307/3801967

[ece35179-bib-0058] Woodbridge, B. , Hansen, D. L. , & Dunk, J. R. (2012). Northern goshawk in California: a technical assessment of its current status and ecology.

[ece35179-bib-0059] Wynne, K. M. , & Sherburne, J. A. (1984). Summer home range use by adult marten in northwestern Maine. Canadian Journal of Zoology, 62, 941–943. 10.1139/z84-132

[ece35179-bib-0060] Ydenberg, R. C. , Brown, J. S. , & Stephens, D. W. (2007). Foraging: an overview In Stephens D. W., Brown J. S., & Ydenberg R. C. (Eds.), Foraging: behavior and ecology (pp. 1–28). Chicago, IL: Chicago University Press.

[ece35179-bib-0061] Zielinski, W. J. , Slauson, K. M. , Carroll, C. R. , Kent, C. J. , & Kudrna, D. G. (2001). Status of American martens in coastal forests of the Pacific states. Journal of Mammalogy, 82, 478–490. 10.1644/1545-1542(2001)082<0478:SOAMIC=2.0.CO;2

[ece35179-bib-0062] Zielinski, W. J. , Spencer, W. D. , & Barrett, R. D. (1983). Relationship between food habits and activity patterns of pine martens. Journal of Mammalogy, 64, 387–396. 10.2307/1380351

[ece35179-bib-0063] Zielinski, W. J. , Truex, R. L. , Schlexer, F. V. , Campbell, L. A. , & Carroll, C. (2005). Historical and contemporary distributions of carnivores in forests of the Sierra Nevada, California, USA. Journal of Biogeography, 32, 1385–1407. 10.1111/j.1365-2699.2005.01234.x

